# Arytenoideraphy—a New Surgical Treatment for Roaring

**Published:** 1898-09

**Authors:** L. A. Merillat

**Affiliations:** Chicago, Ill.


### REPORTS OF CASES.

### ARYTENOIDERAPHY—A NEW SURGICAL TREATMENT FOR ROARING.

By L. A. Merillat, V.S.
CHICAGO, ILL.

*History.* This new surgical procedure originated at the surgical clinics of the McKillip Veterinary College during the session of 1897–1898. The first operation was performed November 18, 1897. It was first advocated by Dr. M. H. McKillip, Dr. W. E. A. Wyman, and the writer.

On account of the low percentage of recoveries, arytenectomy had long since been excluded from the category of surgical operations at the clinics, but the continual appearance of such cases induced the department of surgery to undertake a series of experiments along new lines.

During these experiments the idea was conceived to fasten the arytenoid cartilage to the lateral wall of the larynx, so as to increase its lumen to a degree equal to its lumen during a forcible inspiration. This was done by pinning it to the thyroid cartilage by means of a silver wire. This simple procedure proved to give only temporary relief, as the presence of the wire caused hypertrophy of the cartilage to such an extent as to obstruct the passage quite as much as before the operation. Various other sutures applied in the same manner proved to give the same results. Examination of the parts in each case ten, twenty, and thirty days after showed the arytenoid cartilage dangerously enlarged. It was, therefore, evident that sewing through the body of the cartilage must be abandoned.

In the next experiment the vocal cord was excised and the cartilage fastened outward by passing a suture, not through the cartilage itself, but through the mucous membrane at its very edge at the point where the vocal cord is attached. The operation performed under general anæsthesia, with the patient in the dorsal position, is divided into three steps, as follows :

1. Laryngotomy, or opening the larynx so as to expose its internal aspect. The incision through the integument and muscles is made slowly and carefully and all hemorrhage arrested before making the laryngeal incision. The latter incision is confined to the cricoid cartilage and the crico-thyroidean membrane, or in other words, from the first tracheal ring to the thyroid base (Pommum Adami).

2. Excision of the vocal cord. With the retractors the incision is carefully dilated, the cord pulled upward with a tenaculum, and excised with a curved scissors.

3. Arytenoideraphy proper. A strong silk suture armed with a curved needle is passed through the very edge of the cartilage at the insertion of the vocal cord, then through the crico-thyroidean ligament and back to the point of insertion. Firmly tying the suture in a double surgeon's knot completes the operation.

